# Basic and complex cognitive functions in Adult ADHD

**DOI:** 10.1371/journal.pone.0256228

**Published:** 2021-09-02

**Authors:** Saleh M. H. Mohamed, Marah Butzbach, Anselm B. M Fuermaier, Matthias Weisbrod, Steffen Aschenbrenner, Lara Tucha, Oliver Tucha

**Affiliations:** 1 Department of Clinical and Developmental Neuropsychology, University of Groningen, Groningen, Netherlands; 2 Psychiatry and Psychotherapy, SRH Clinic Karlsbad-Langensteinbach, Karlsbad, Germany; 3 General Psychiatry, Center of Psychosocial Medicine, University of Heidelberg, Heidelberg, Germany; 4 Clinical Psychology and Neuropsychology, SRH Clinic Karlsbad-Langensteinbach, Karlsbad, Germany; 5 Department of Psychiatry and Psychotherapy, University Medical Center Rostock, Rostock, Germany; 6 Department of Psychology, Maynooth University, National University of Ireland, Maynooth, Ireland; University of Oviedo, SPAIN

## Abstract

**Background:**

Many clinical studies reported deficits in basic and complex cognitive functions in adults with Attention-Deficit/Hyperactivity Disorder (ADHD). However, the extent in which deficits in basic functions (i.e., processing speed and distractibility) contribute to complex cognitive impairments (i.e., working memory, planning, cognitive flexibility, memory functions) in adults with ADHD is not well-studied. So far, literature show only one study, revealing that basic functions explain 27–74% of executive dysfunctions. Yet, the authors reported that findings could be affected by the selection of neuropsychological tests. The goal of the present research is to replicate such a finding using a different sample and a different set of neuropsychological tests.

**Methods:**

Forty-eight adult patients with ADHD were compared with 48 healthy controls in basic cognitive functions, namely processing speed and distractibility and more complex cognitive functions, namely selective attention, cognitive flexibility, planning, working memory, verbal fluency, and verbal memory. Basic and complex cognitive functions were assessed using the Vigilance and Sustained Attention, Selective Attention, N-Back, Tower of London, Trail Making Test, Word Fluency, and Verbal Learning and Memory.

**Results and conclusion:**

Logistic regression analyses showed that impairments in complex cognitive functions explained 25% of the variance in ADHD diagnosis. The explained variance dropped from 25% to 9% after considering basic functions of processing speed and distractibility. This 64% reduction highlights the importance of basic functions for impairments in complex functions in patients with ADHD.

## Introduction

Attention-deficit/hyperactivity disorder (ADHD) is a developmental disorder that negatively affects several life domains. Individuals with ADHD have a higher risk to experience academic and occupational difficulties, and problems in their interpersonal relationships relative to their peers [1–3]. The main behavioral symptoms of the disorder, as described in the Diagnostic and Statistical Manual of Mental Disorders (DSM), are manifestations of inattention, hyperactivity, and impulsivity, which start in early childhood and persist (especially the inattentive symptoms) into adulthood in about two thirds of the diagnosed cases [4].

Besides the behavioral symptoms of ADHD, studies have reported impairments in basic cognitive processes such as slow processing speed, distractibility, and increased reaction time variability [5–9]. Processing speed refers to how quickly an individual can react to a given stimulus within a limited time frame, it does not reflect individual differences in specific abilities, but rather differences in the time needed to execute cognitive operations [10]. Distractibility refers to individuals’ attention being pulled away from the target stimulus [5]. More specifically, the shift in attention toward the non-target stimulus possibly leads to incomplete or incorrect encoding of the target stimuli (e.g., missing a go signal/cue required to execute a correct response in a stop signal task). Increased distractibility in ADHD is attributed to an inability to filter out irrelevant information [11] or excessive orientation towards task-irrelevant stimuli [12]. Dysfunctions in cortico-striato-thalamo-cortical neuroanatomical circuitry are thought to produce periodic lapses of attention causing periodic fluctuations and increased reaction time variability in children with ADHD [13]. Increased reaction time variability, in particular, is a consistently replicated deficit of neuropsychological performance in ADHD [5,14,15]. Furthermore, compared to other aspects of task performance (e.g., reaction time delay and error rate), increased reaction time variability has been the most robust finding. The studies even suggested that slow processing speed in ADHD may disappear after controlling for reaction time variability ADHD [5,14,15]. In most of the previous studies processing speed was measured by mean reaction times to simple stimuli; while distractibility was measured by reaction time variability and/or omission errors, for review on reaction time variability in ADHD see [5,16,17].

Impairments in complex cognitive functions such as executive functioning and memory have also been reported in ADHD [18–20], for a meta-analysis see [21]. As for basic cognitive processes, many of complex cognitive functions are commonly indexed by slower and/or less accurate responses in different laboratory tasks such as working memory tasks [22], Stroop and Go/No-go response inhibition tasks, measuring executive functions [23,24].

Basic cognitive processes can be seen as a foundation of complex cognitive processes [25]. For example, when an individual shows a deficit in reaction time and errors as a result of increased reaction time variability [15], and being easily distracted (i.e., deficits in basic processes), these deficits may manifest in any more complex function that built up on these processes. Put differently, the measured task performance is an outcome of both basic and complex cognitive processes combined. The questions emerge to what extent each type of these processes independently contribute to poor task performance in ADHD and whether tasks’ indices, used to estimate complex cognitive functions reflect impairments in basic information processing rather than deficiencies in complex cognitive processing.

The above-mentioned questions have been partly addressed in children with ADHD. Metin et al. [26] and Salum et al. [27] showed that reaction time and performance accuracy combined reflect inefficient basic information processing rather than independent effects of executive dysfunctions in children with ADHD. This is also in line with previous findings showing that slow processing speed might cause poor working memory in ADHD [28]. Inefficient basic processing in ADHD has been observed, among others, in a slow accumulation rate of relevant information needed for a correct response decision during both low and high demanding cognitive tasks [26,27]. The slow rate of information accumulation depicts difficulties in detecting signals from noise and has been found to account for increased reaction time variability [29] and may also explain slow reaction times and increased number of errors in ADHD performance. A more recent study by Caspersen and colleagues [30] has elaborated on such an inefficiency in basic processes in children with ADHD by disentangling perceptual and response-based deficits, suggesting that processing inefficiency occurs at an early perceptual level as evident by slow visual processing speed and correlated increased omission errors on tests targeting perceptual functions in children with ADHD.

In adolescents and adults with ADHD the topic received less attention, despite the overwhelming evidence documenting basic impairments in processing speed and distractibility [5,14,15,31–33]. A systematic review and meta-analysis by Cook and colleagues [34] on processing speed and its associations with clinical and functional outcomes has revealed that processing speed appears to be associated with reading abilities and complex adaptive functioning skills in adolescents with ADHD. The latter underlines the vital role of basic processes in adaptation to and performance of complex tasks that require ongoing higher order (top-down) executive control.

In a recent study, Butzbach et al. [25] have directly examined the contribution of basic processes to complex cognitive impairments in adults with ADHD. The authors made a distinction between basic and complex cognitive functions based on the demands of the tasks and their levels of complexity. Processing speed and distractibility were respectively measured by the mean reaction times and both standard deviations and omission errors on a simple alertness and a vigilance task. Complex cognitive functions were measured using more complex tasks such as the Stroop test and the Trail Making Test (trial B). Results showed that processing speed and distractibility could respectively explain 41% and 43% of the impairments on executive function tasks, 29% and 27% of the impairments of memory functions, and 56% and 74% of the impairments of complex attention. The results indicated that basic processes constitute a significant proportion of complex cognitive impairments in adults with ADHD. However, the authors reported that findings could be affected by the selection of neuropsychological tests, guiding future studies to replicate the study using different neuropsychological tests.

In response, using a different adult sample and a different test selection, the present study aims to replicate Butzbach and colleagues work in adults with ADHD [25], following their approach to differentiate basic from complex cognitive functions. More specifically, basic functions were measured using a low demanding task regarding the complexity level of stimuli (e.g., presenting simple stimuli with no or minimum number of distracting features) and required responses (e.g., giving one response to a simple visual stimulus; while complex functions were measured using more demanding tasks with increased complexity level of both stimuli and required responses.

The contribution of the present study to the literature can be explained as it follows: Butzbach et al. [25] reported novel and original outcomes, yet it is possible that these outcomes might be influenced by nonreplicable artifacts. For example, the outcomes might be limited by certain characteristics of the sample tested in Butzbach et al. study [25], the presentation of comorbidities, and/or the employed neuropsychological tests. It is well-known that ADHD is heterogeneous disorder with diverse neuropsychological impairments and comorbid symptomatology. This points towards the value of the present study, which attempts to enhance the generalizability and the validity of original outcomes [25]. Block and Kuckertz [35] highlighted the importance of conducting replication studies. For example, replication studies can indicate whether a research finding is robust, despite variance in testing situations. In addition, there is a scarcity of studies testing the role of basic functions in complex cognitive performance in adults with ADHD. More studies based on clinical populations are needed to understand the nature and manifestations of cognitive impairments in adults with ADHD.

Briefly, the present study aims to examine the following:

1. the extent to which basic functions (i.e., processing speed and distractibility) and complex cognitive functions (i.e., executive functions, verbal memory, and selective attention) are impaired in adults with ADHD, compared to healthy controls.
2. the extent to which impairments in complex cognitive functions contribute to variance in adult ADHD diagnosis when impairments in basic cognitive functions are considered.

Based on the abovementioned literature, see for example [5,14,21], adults with ADHD are expected to demonstrate impairments in basic functions (namely, slower processing speed and increased distractibility) as well as impairments in more complex cognitive functions (namely, difficulties in selective attention, working memory, planning, cognitive flexibility, verbal fluency, and memory functions), compared to healthy controls. Findings of the previous work [25] indicate that impairments in basic functions may explain a considerable proportion of complex cognitive impairments in adults with ADHD. Accordingly, impairments in complex cognitive functions are expected to explain lower variance in ADHD diagnosis when basic functions are considered.

## Method

### Participants

Fifty-four adults diagnosed with ADHD were recruited from the SRH hospital, Karlsbad-Langensteinbach, Germany. Each individual in this patient group underwent extensive diagnostic assessment that included a clinical interview based on the DSM-IV ADHD diagnostic criteria. ADHD symptoms, perceived cognitive impairments, and related functional impairments of the patient group were also assessed using the Conners’ Adult ADHD Rating Scales (CAARS) [36], the Weiss Functional Impairment Rating Scale (WFIRS) [37,38], and the Questionnaire for Complaints of Cognitive Disturbances (FLei) [39]. To control for occurrence of noncredible symptom report and performance in adults with ADHD, individuals were excluded if they failed both a measure of symptom validity (the CAARS Infrequency Index (CII) [40], and performance validity on the Groninger Effort Test (GET) [41]). Based on this criterion, three individuals from the patient group were excluded. Another three patients were excluded due to disruptions of the testing situation. For the remaining patient sample (i.e., 48 patients), there were 21 patients had Inattentive subtype of ADHD, one patient had Hyperactive-Impulsive subtype, and 26 patients had Combined subtype (please note, classification of ADHD subtypes was based on the 10th revision of the international statistical classification of diseases and related health problems (ICD). Table 1 provides clinical and descriptive information, including information about age, gender, IQ as measured by scores on a test for vocabulary skills (i.e., Multiple Choice Vocabulary Test (MWT-B) [42]), education level, education years, ADHD subtypes, and other co-existing disorders. Education level was measured by asking the participants to report the highest degree they have obtained. With regard to medication, five patients received stimulants, fourteen patients received antidepressants, and seven received medications for other disorders such as epilepsy.

**Table 1:** Characteristics of 48 patients with ADHD and 48 healthy controls.

	Patients with ADHD (*Mean ± SD*)	Healthy controls (*Mean ± SD*)	Effect size (*r*)	P-value
Age (years)	36.10 ± 11.16	34.15 ± 12.60	-	.403
Gender	20 females, 28 males	26 females, 22 males	-	-
Education level ^a^	1.85 ± 1.35	1.04 ± 1.05	-.30	.003
Education years	16.21 ± 3.64	17.20 ± 2.89	-.16	.123
Intellectual functions (scores on MWT-B)	104.90 ± 11.39	108.32 ± 13.05	-.09	.358
Co-existing disorders ^b^	36 patients	-	-	-
Average score on the Flei	24.35 ± 7.21	10.33 ± 5.37	-.73	.000
Average score on the WFIRS	1.14 ± 0.48	0.50 ± 0.30	-.64	.000
DSM-IV Total ADHD Symptom subscale of the CAARS	26.64 ± 8.38	11.06 ± 6.43	-.74	.000

Note. ADHD = Attention-Deficit/Hyperactivity Disorder; MWT-B = The German multiple-choice Vocabulary test; FLei = Questionnaire for complaints of cognitive disturbances; WFIRS = Weiss Functional Impairment Rating Scale; CAARS = Conners’ adult ADHD rating scales.

^a^ Educational level was measured as a nominal variable with the following numeric codes: 0 = University education,1 = German Abitur (e.g., highest secondary education), 2 = Fachhochschule (e.g., university of applied sciences),3 = Realschule (e.g., middle secondary education), 4 = Hauptschule (lowest secondary education), 5 = no educational degree.

^b^ there were 20 comorbid disorders, examples of such disorders are depression, anxiety, multiple substance abuse, psychosomatic complaints, panic disorder, epilepsy, and obsessive–compulsive disorder.

The control group was recruited through public advertisements. We initially considered 104 participants for the inclusion to the control group before we excluded individuals who report a diagnosis of psychiatric disorders. Next, 48 controls were selected case by case to match the patient group in age, IQ and educational years. A nonparametric comparison (i.e., Mann-Whitney tests) revealed no significant differences between patients with ADHD and controls in age and educational years. However, patients with ADHD had a significantly lower level of educational attainment than controls. Table 1 shows the characteristics (means and standard deviations) of the patient and control group. The control and patient groups were compared in self-reported ADHD symptoms, daily functional impairments and cognitive complaints as measured, respectively, by the DSM-IV Total ADHD Symptoms subscale of the CAARS, the total scores of the WFIRS and the total score of the FLei. Mann-Whitney tests showed that, compared to the control group, patients with ADHD had significantly higher scores on the three scales.

### Measures

The CAARS, WFIRS, and FLei scales were used to measure ADHD symptoms and related functional impairments. The CII scale and the GET test were applied to determine noncredible symptom report and performance. In addition, five subtests from the Vienna Test System (VTS) [43] were used to measure processing speed, distractibility, working memory, planning, and cognitive flexibility (using the vigilance and sustained attention, selective attention, N-Back, Tower of London, and Trail Making Test, respectively). The VTS is a testing system of computerized tests measuring a variety of neuropsychological functions. Verbal memory and verbal fluency are measured by the Verbal Learning and Memory Test [44] and the Regensburg Word Fluency Test [45], respectively. Below, detailed description of all scales and tests used in this study.

#### Conners’ Adult ADHD Rating Scales (CAARS)

The CAARS [36] was administrated to measure adult ADHD symptoms. Participants rated their symptoms on a 4-point Likert-type scale ranging from 0 (‘not at all, never’) to 3 (‘very much, very frequently’). The CAARS contained, among other subscales, two subscales that assessed ADHD symptoms listed in the Diagnostic and Statistical Manual of Mental Disorders-the fourth edition (DSM-IV), namely the Inattentive Symptoms subscale and the Hyperactive-Impulsive Symptoms subscale. The sum scores of these two subscales represented scores on a third subscale, called the ADHD Symptoms Total. Psychometric properties of the CAARS were found to be satisfactory, with an internal consistency of.86 -.92 for the four main dimensions and test-retest reliability of.80-.91 across the different subscales. Further, a sensitivity to ADHD symptoms of 82% was found along with a specificity of 87% [46].

#### CAARS Infrequency Index (CII)

The CII [40] was developed as a subscale of the CAARS to identify noncredible reports of ADHD symptoms. The CII consisted of items that were infrequently endorsed by healthy individuals and genuine patients with ADHD, and thus high scores on this scale might indicate over-reporting or noncredible symptom report. The subscale demonstrated moderate sensitivity to extreme scores on the Inattentive Symptoms and Hyperactive-Impulsive Symptoms subscales of the CAARA. A cut-off score of 21 or higher on the CII was proven to have excellent specificity and thus was taken as a standard [40]. Consequently, scores equal or above 21 on the CII in the present study were considered to reflect noncredible responses on the CAARS.

#### Groningen Effort Test (GET)

The GET [41] was designed as a computerized visual discrimination task to detect non-credible performance of those who feign ADHD. The task might appear demanding in terms of concentration and attention allocation. In contrary to what the task appeared to be, patients with ADHD easily performed the task as most of healthy individuals did [41]. As such, noncredible test takers were expected to have more errors than those with genuine diagnosis of ADHD. In addition, task instructions put emphasis and reminded participants about the supposedly demanding nature of the task during testing. The task showed high accuracy in distinguishing between credible and non-credible performance with 89% sensitivity and 89.5% specificity [41].

#### Weiss Functional Impairment Rating Scale (WFIRS)

The WFIRS [37,38] was used to estimate impairments related to ADHD in seven different life settings: namely family, work, college, general life skills, self-concept, social functioning, and risk taking. The WFIRS consisted of 70 items on a 4-point scale (scored from 0 = never or not at all to 3 = very often or very much). Psychometric characteristics of the WFIRS were good [38]. Cronbach’s alpha coefficients for the Family, Work, College, Life Skills, Self-concept, Social Functioning, and Risk subscales were reported to be.86,.91,.90,.89,.94,.88, and.88, respectively [47].

#### Questionnaire for Complaints of Cognitive Disturbances (FLei)

The Flei [39] was a self-report scale developed in the German language, measuring cognitive complaints in attention, memory, and executive functioning in daily situations. The Flei consisted of 30 items on a 4-point scale from 0 (never) to 4 (very frequently). Three subscales of the FLei were used, namely Attention, Memory and Executive Functions. A total score was computed out of the three subscales. Higher total scores indicated more cognitive complaints. The scale demonstrated good split-half reliability and high consistency [39].

#### Vigilance and Sustained Attention Test (WAFV)

The WAFV is a subtest of the VTS [48] and was used to measure processing speed and distractibility. The WAFV included a simple visual stimulus (i.e., a square), which changed its intensity of grey shading for some trials. The stimulus was presented for 1500 ms in the center of the computer screen at regular intervals (500 ms interstimulus interval). After each 500 ms presentation time, the color of the square (i.e., the target stimulus) may get darker. Participants had to detect whether the target stimulus had turned darker or not by pressing a specific button on the response device as fast as possible. The short version of the WAFV was used. In the test, there was about one target stimulus per minute and fifteen changes in color per testing session. Participants were asked to remain alert and ready to react to infrequently occurring target stimuli. The mean reaction time, logarithmic standard deviation of the reaction times (dispersion of reaction time across conditions), and the number of omission errors were registered. The mean reaction time estimated processing speed, while both the dispersion of reaction time and omission errors measured distractibility.

Using factor analysis, the construct validity of the WAFV was checked with other tests, namely the Cognitrone [49], Discrimination, and Reaction [43] tests for convergent validity, and the Standard Progressive Matrices test [50] for discrimination validity. Results showed that the WAFV had an adequate convergent validity as the WAFV and those above-mentioned tests load onto the same factor. In addition, the WAFV was distinguished from non-verbal intelligence supporting the discrimination validity of the WAFV [48]. The reliability of the WAFV was also investigated and yielded a very good internal consistency, Cronbach’s alpha coefficients ranged from.96 to.99 [48].

#### Selective Attention Test (WAFS)

The WAFS [48] was another subtest of the VTS, which was used to evaluate the ability to selectively pay attention to specific features of presented stimuli. In this test, participants saw visual shapes (circles, squares or triangles) in the center of the screen. Each stimulus started with a shape presented for 500 ms. Next, the same shape (i.e., the target stimulus) was presented for 1000 ms. either in the same or different shading (i.e., lighter or darker). The interstimulus interval was 1000 ms. The WAFS subtest consisted of 475 shapes, which were presented in pseudorandomized order and out of these shapes, there were 100 changes in the shading of the stimulus. Participants were asked to pay attention to the change of shading of circles and squares only (i.e., target stimuli). Participants were asked to ignore changes in triangles (i.e., distractors). Once participants detected a change, they had to press a button on a response panel. Errors of commissions (that included responses to distractors and responses to stimuli without a change in shading) were used to index selective attention.

The construct validity of the WAFS was investigated [48], results showed that the WAFS had acceptable convergent and discrimination validity. The WAFS showed also a very good internal consistency, Cronbach’s alpha coefficients ranged from.94 to.97 [48].

#### N-Back test

The 2-back VTS subtest was used to measure working memory [51]. During the test, 100 consecutive letters were displayed on a screen, one letter at a time. Each letter was presented for 1500 ms, followed by an inter-stimulus interval of 1500 ms. Participants were asked to decide whether the currently presented letter matched a previously presented letter which was presented two places back (i.e., two-back condition). The decision was made by pressing a button from the response panel if there was a match, otherwise no response was required. Scores on the 2-back test were calculated as the total number of correct responses. However, the scores were inverted, meaning that higher scores reflected more problems in working memory.

Although the construct validity of the n-back test has been questioned as it showed no or weak correlations with other working memory tests (such as reading span task and digit span task, see for example a study by Jaeggi and colleagues [52], there were several indications supporting the validity of the N-back test [51]. For instance, performing the N-back test activated a neural network related with working memory, monitoring, inhibition and rehearsal [51]. Regarding the reliability, the N-back test revealed Cronbach’s alpha coefficients between.85 to.89.

#### Tower of London-Freiburg Version (TOL)

The TOL of the VTS [53] was used to assess planning ability. The test was computerized, wherein a wooden model containing balls was displayed on a screen. In the model, the left-hand rod was the highest and could hold three balls, the middle rod was shorter and could hold two balls, and the right-hand rod was the shortest rod and could hold one ball. There were three balls in the following colors: red, yellow or blue. The test consisted of 28 goal states, each had a different distribution of the balls among rods. For each item, there was a starting distribution state and a target distribution state. Participants were asked to reach the target configuration by using a computer mouse to transfer the balls among the rods with minimum number of moves and within 60 seconds. While moving the balls, the three following rules had to be followed: (1) The balls should not be placed on rods that were fully occupied with balls, (2) Balls that were blocked by balls lying on top of them could not be selected, and (3) Only one ball could be moved at a time. After 60 seconds, the item that was being worked on was automatically terminated. The TOL was terminated if three items in succession were not completed successfully [53]. The planning ability was estimated by calculating number of items which were correctly solved in a minimum number of moves. Errors were thus excluded from calculating the scores. Scores on the TOL were inverted, higher inverted scores indicated poor planning abilities.

Köstering et al [54] discussed that the test demonstrates a good criterion validity in revealing disease-related deficits in planning performance. The internal consistency was adequate with Cronbach’s alpha coefficients above.70 [53].

#### Trail Making Test-Langensteinbacher Version (TMT)

The TMT of the VTS [55] had two trials: Trial A and Trial B. The Trial A measured processing speed; while Trial B evaluated cognitive flexibility and attentional switching. In the Trial A, numbers were arranged pseudo-randomly on the screen. Using the mouse device with the dominant hand, participants should connect numbers (1–25) in ascending order. In the Trial B, both numbers (1–13) and letters (A-L) were arranged pseudo-randomly on the screen. Participants were asked to connect numbers with letters and to alternate between them one at a time, so that at the end of the Trial B the connected line should contain numbers and letters sequences that were respectively ordered in an ascending and alphabetic way (i.e., 1-A-2-B-3-C-4-D, etc.). Before each trial, participants received one practice sample of each trial, participants were instructed to connect numbers or numbers with letters as quickly as possible.

For Trial A, scores were calculated by counting the number of seconds required to complete the trial. For Trial B, cognitive flexibility was indexed by counting the working time (number of seconds) and dividing it by the working time of the Trial A in order to control for the processing speed. Accordingly, higher scores indicated lower performance.

A number of studies showed that the TMT test, in particular Trial B, had good convergent and discrimination validity to measure cognitive flexibility [55,56]. Using the greatest lower bound [55], the reliability of quotient (Trial B/trial A) was estimated (to be.798) and it was considered acceptable.

#### Regensburg Word Fluency Test (RWT)

The RWT [45] is a standard test for verbal fluency developed in the German language [45]. Participants were asked to produce as many words as possible, starting with the letter ‘M’, while following certain rules. The rules were (1) not to use any kind of name, (2) not to use words that start with the same stem (e.g., bat and batman), (3) not to repeat words, and (4) to use only words found in German news and books. Verbal responses were registered by the examiner. The score was calculated by summing up number of correctly generated words within two minutes. Higher scores represented better functioning, however in the present study, scores were inverted, meaning that higher scores reflected lower abilities for verbal fluency.

The RWT was proven to distinguish between healthy individuals and patients with psychiatric disorders such as Alzheimer’s disease, Parkinson’s disease, and patients with brain injury [45,57]. Test-retest reliability was high (*r* = .81). Scores on a different version of the test (words starting with the letter ‘P’) were adequately correlated with scores on the test (*r* = .671) [45].

#### The Verbal Learning and Memory Tests (VLMT)

In the VLMT [44], the examiner read aloud a list of 15 words (i.e., list A). After reading list A, participants were immediately asked to recall as many words as possible from the list. This was repeated for another four trials. After the fifth trial, the examiner read aloud another list of 15 words (i.e., list B), containing different words than those used in list A. Participants were asked to recall as many words as possible from list B. After recalling list B, they had to recall list A again (without it being read aloud again). Next, a break of 20–30 minutes was administered. During this break, participants were asked to perform two or three non-verbal neuropsychological tests. Participants were not told that after the break they would be asked to recall list A. After the break, participants freely recalled words from list A. Thereafter, as a recognition test, the examiner presented a third list of 50 words, which contained words from list A. The examiner asked participants if they recognized words form the list A.

The test offers four scores: (1) the immediate recall after hearing the list once, (2) sum of correctly recalled words from the first five trials (i.e., verbal learning sum score), (3) number of correctly recalled words from list A after a 20–30 minutes delay (i.e., delayed recall score), and (4) the number of correctly recognized words from list A at the end of the test (i.e., recognition score). Only delayed recall scores are analyzed as they reflect memory capacity and minimize the effect of learning [44]. Scores on the VLMT were inverted, higher scores indicated more impairments in memory.

An early version of the VLMT [58] was able to differentiate between patients with left and right temporal lobe epilepsy: patients with left temporal lobe epilepsy showed poorer delayed free call. However, correlations of the VLMT with other memory tests were low to moderate [44]. Retests with parallel forms of the VLMT revealed correlations between.60 to.77, indicating an acceptable reliability of the VLMT [44].

### Procedure

The study was approved by the ethical committee of the medical faculty of the university of Heidelberg, Germany, as it meets the Helsinki Declaration standards. Participants performed the neuropsychological tests individually. In the beginning of each session, the examiner explained the goal and the structure of the testing (i.e., the order of filling out the questionnaires and what participants needed to do) to the participants. Next, participants were asked to sign the informed consent forms if they agreed with the procedures. Thereafter, participants reported their demographic information and completed the MWT-B (IQ) test, followed by performing the neuropsychological tests. To avoid introducing fatigue or order effects, the order of neuropsychological tests was altered and counterbalanced across participants. Participants were allowed to take short breaks as needed. After completing the neuropsychological tests, participants filled out self-reported scales, namely the CAARS, WFIRS, and FLei. The GET test was administered either before or after the other neuropsychological tests.

Participants were debriefed at the end of the assessment. The testing duration for each participant was approximately 2–3 hours.

### Data analysis

Basic cognitive functions can be defined as simple fundamental cognitive processes such as distractibility and processing speed, which may be prerequisites for task performance. Complex cognitive functions refer to a higher level of cognitive processes such as planning and working memory, which rely on the combination and interaction of more basic functions [59]. Based on the demands of the tasks, basic and more complex cognitive functions were operationally differentiated: basic functions were measured from a non-demanding simple task (i.e., the WAFV that includes a simple stimulus presentation with minimized distracting features and one simple response); while more complex functions were measured using more demanding tasks with increased level of complexity of the presented stimuli and required responses (i.e., the WAFS, TOL, TMT, TOL, RWT, and VLMT tests).

The basic functions assessed in the present study were processing speed and distractibility: processing speed was measured by mean reaction times on the WAFV. Distractibility was measured by both the mean dispersion of reaction time (i.e., logarithmic standard deviation of reaction times) and number of omission errors in the WAFV. The complex cognitive functions assessed were selective attention, cognitive flexibility, planning, working memory, verbal fluency, and verbal memory, which were measured respectively by number of commission errors on the WAFS, time of Trial B/time of Trial A on the TMT, number of correct responses on the TOL, number of correct responses on the 2-back, number of correctly generated words within two minutes in the RWT, and number of correctly recalled words in the delayed recall phase of the VLMT.

Scores on the TOL, RWT, N-back, and VLMT tests were inverted by multiplying them by (-1). This inversion aims to unify the interpretation of scores from different tests used in the present study: the higher score is, the greater impairment in the measured cognitive ability.

SPSS software (version 26) was used to run all statistical tests in the study. The data was checked for normality. The Shapiro-Wilk tests showed that data deviated from the normal distribution for the TMT, N-back, TOL, VLMT, WAFS, omission errors and standard deviations on the WAFV tests (*p* ≤.006); while no indication of a deviation from the normal distribution was found for mean reaction times on the WAFV and scores on the RWT (*p* ≥.084). Thus, other assumptions for parametric testing such as homogeneity of variance were not checked further. Because the data violated the normality assumption of t-tests, Mann-Whitney tests were performed to test differences between patients and healthy controls in basic and more complex cognitive processes. Due to multiple comparisons and possible inflation of alpha error, the acceptable p-value was set to.005 instead of.05, following the Bonferroni correction. In addition, effect sizes for non-parametric Mann-Whitney tests were calculated using the following formula: (*r = z/square root of N*) where *N* represents the total number of cases (see Fritz, Morris, and Richler (2012) and Rosenthal (1991)). Interpretations of effect sizes were based on Cohen’s criteria [60], namely 0.1 = small effect, 0.3 = medium effect, and 0.5 = large effect.

To examine the extent to which basic and cognitive functions are impaired in adults with ADHD compared to healthy controls, Mann-Whitney tests were performed to test group differences in all variables (i.e., mean reaction time on the WAFV, reaction time variability on the WAFV, number of omission errors in the WAFV, number of commission errors on the WAFS, time of Trial B/time of Trial A on the TMT, number of correct responses on the TOL, number of correct responses on the 2-back, number of correctly generated words within two minutes in the RWT, and number of correctly recalled words in the delayed recall phase of the VLMT).

To investigate the contribution of complex cognitive functions to variance in ADHD diagnosis, a logistic regression was performed. The dependent variable was a binary group variable (the control group versus the patient group). The independent variables were all test variables of complex cognitive functions. These independent variables were implemented in one model (step) to test whether the probability of group membership (i.e., an estimation of how likely the case is to belong to each group) can be predicted from scores on the abovementioned set of neuropsychological tests. The data fulfilled the assumptions of logistic regression as evident by the Hosmer & Lemeshow test (*X*^*2*^ (*df*) = 5.71 (8), *p* = .679) and the Omnibus test (*X*^*2*^ (*df*) = 19.23 (6), *p* = .004). The Hosmer and Lemeshow test is a goodness of fit test, which describes how well data fits the statistical regression model. Specifically, it calculates whether the observed values do not match the expected values from the model, suggesting poor predictions and lack of fit. Here, low p-values indicate that the model should be rejected. The omnibus test indicates whether the new model describes group membership better than pure chance. Put differently, the test indicates whether the inclusion of a block of variables is significantly better than a model with only the intercept.

To test the contribution of impairments in complex cognitive functions to variance in adult ADHD diagnosis after considering impairments in basic cognitive functions, a hierarchical logistic regression was performed. In the first model, measures of basic cognitive processes were entered as predictors. In the second model, measures of complex cognitive processes were entered as predictors. The dependent variable was a binary group variable (the control group versus the patient group). The data fulfilled the assumptions of logistic regression as confirmed by the Hosmer & Lemeshow test (*X*^*2*^ (*df*) = 14.05 (8), *p* = .080 for the first model and *X*^*2*^ (*df*) = 4.99 (8), *p* = .758 for the second model) and the Omnibus test (*X*^*2*^ (*df*) = 20.22 (3), *p* = .000 for the first model and *X*^*2*^ (*df*) = 28.10 (9), *p* = .001 for the second model).

## Results

### Group differences in basic and more complex cognitive functions

Table 2 presents means and standard deviations on all neuropsychological tests. Regarding basic cognitive functions, Mann-Whitney tests revealed significant differences between patients with ADHD and controls in mean reaction times and omission errors on the WAFV test with medium effect sizes (see Table 2): patients were slower and showed more omission errors compared to controls. However, no significant group differences were found in reaction time variability during the WAFV.

**Table 2:** Scores on the neuropsychological tests in 48 adult patients with ADHD and 48 healthy controls.

		Patients with ADHD (*Mean ± SD)*	Healthy controls (*Mean ± SD)*	Effect size (*r*)	P-value
Basic functions	WAFV: Mean reaction times	444 ± 84	384 ± 81	-.35	.001*
	WAFV: Omission errors	1.83 ± 2.69	0.32 ± 0.73	-.39	.000*
	WAFV: Logarithmic standard deviation of reaction times	1.26 ± 0.12	1.22 ± 0.99	-.20	.053
Complex functions	WAFS: Commission errors	4.23 ± 4.00	1.81 ± 1.77	-.34	.001*
	TMT: The cognitive flexibility index scores	1.77 ± 0.56	1.61 ± 0.52	-.17	.094
	TOL scores ^b^	-14.93 ± 3.06	-15.79 ± 3.89	-16	.113
	N-Back scores ^b^	-11.30 ± 2.74	-12.17 ± 3.31	-.21	.036
	RWT scores ^b^	-16.06 ± 4.50	-17.96 ± 5.76	-.18	.078
	VLMT-delayed recall scores ^b^	-11.06 ± 2.79	-12.55 ± 2.57	-.29	.005*

Note WAFV = Vigilance and Sustained Attention Test; WAFS = Selective Attention Test; TMT = Trail Making Test; TOL = Tower of London Test; RWT = Regensburg Word Fluency Test; VLMT = Verbal Learning and Memory Test.

^b^ inverted scores (scores × -1).

* significant (p-values were set to 005 or below).

For complex cognitive functions, the two groups significantly differed in scores on the WAFS and VLMT test with medium effect sizes (see Table 2): patients with ADHD committed more errors on the WAFS test and showed poorer performance on the VLMT delayed recall compared to controls. Scores of the Cognitive Flexibility Index, RWT, N-back, and TOL tests were not significantly different between the two groups (see Table 2).

### The contribution of basic processes to more complex cognitive processes in predicting group membership

Table 3 shows that basic cognitive functions explained about 27% of the group differences (*Nagelkerke R*^*2*^ of model 1 = .27). Adding complex cognitive functions to this model increased the explained group differences by 9% (*Nagelkerke R*^*2*^ of model 2 (.358) minus *Nagelkerke R*^*2*^ of model 1 (.27) = .09). Without controlling for basic cognitive functions, complex cognitive functions explained about 25% of the group differences (*Nagelkerke R*^*2*^ = .25, see Table 4).

**Table 3:** Hierarchical logistic regression analysis of variables of basic cognitive functions (model 1) and complex functions (model 2) to predict group membership (48 patients with ADHD vs 48 healthy controls).

	Predictors	β	SE	Wald X^2^	P-value	Exp(β)
Model 1 Cox and Snell R^2^ = .20 Nagelkerke R^2^ = .27	WAFV: Mean reaction time	0.00	0.00	1.95	.162	1.00
	WAFV: Logarithmic standard deviation of reaction times	2.22	2.57	0.75	.387	9.22
	WAFV: Omission errors	0.57	0.29	3.96	.047	1.77
Model 2 Cox and Snell R^2^ = .27 Nagelkerke R^2^ = .36	WAFV: Mean reaction time	0.00	0.00	1.66	.197	1.00
	WAFV: Logarithmic standard deviation of reaction times	1.94	2.80	0.48	.489	6.96
	WAFV: Omission errors	0.36	0.30	1.41	.235	1.43
	WAFS: Commission errors	0.25	0.15	2.83	.093	1.28
	TMT: The cognitive flexibility index	0.62	0.51	1.44	.230	1.85
	TOL scores ^b^	-0.06	0.09	0.49	.482	0.94
	N-back scores ^b^	-0.10	0.11	0.95	.329	0.90
	RWT scores ^b^	-0.03	0.06	0.29	.587	0.97

Note Values were approximated to two decimals except for p-values; WAFV = Vigilance and Sustained Attention Test; WAFS = Selective Attention Test; TMT = Trail Making Test; TOL = Tower of London Test; RWT = Regensburg Word Fluency Test; VLMT = Verbal Learning and Memory Test.

^b^ inverted scores (scores × -1).

**Table 4:** Logistic regression analysis of variables of complex cognitive functions in predicting group membership (48 patients with ADHD vs. 48 healthy controls).

	Predictors	β	SE	Wald X^2^	p-value	Exp(β)
The model Cox and Snell R^2^ = .19 Nagelkerke R^2^ = .25	WAFS: Commission errors	.32	.12	6.63	.010	1.37
	TMT: The cognitive flexibility Index	.65	.47	1.90	.168	1.92
	TOL scores	-.04	.08	0.27	.606	0.96
	N-back scores ^b^	-.04	.09	0.23	.634	0.96
	RWT scores ^b^	.00	.05	0.00	.985	1.00
	VLMT-delayed recall scores ^b^	.12	.10	1.57	.210	1.13

Note Values were approximated to two decimals except for p-values; WAFV = Vigilance and Sustained Attention Test; WAFS = Selective Attention Test; TMT = Trail Making Test; TOL = Tower of London Test; RWT = Regensburg Word Fluency Test; VLMT = Verbal Learning and Memory Test.

^b^ inverted scores (scores × -1).

Taken altogether, the contribution of complex functions dropped from.25 to.09 after controlling for basic functions. Consequently, basic functions may account for approximately 64% (*(*.*25-*.*09)/*.*25)*: *Subtracting the increase in R*^*2*^ *(*.*09) when adding complex functions from R*^*2*^ *(*.*25) of the model that includes only complex functions and divide the outcome value by R*^*2*^ *of the model that includes only complex functions*) of complex cognitive functions when explaining differences between patients with ADHD and healthy controls.

## Discussion

In line with ADHD literature [5–9,25], results indicated that patients with ADHD had compromised basic processes as demonstrated by slow processing speed (effect size, *r* = -.35) and increased level of distractibility (effect size, *r* = -.39), effect sizes were medium. However, the groups did not differ in reaction time variability. Patients with ADHD also showed impairments in selective attention (effect size, *r* = -.34), and memory functions (effect size, *r* = -.29), but not in planning, working memory, cognitive flexibility and verbal fluency. Previous studies revealed inconsistent results regarding group differences between adults with ADHD and controls in various executive functions. For example, some studies reported deficits in patients with ADHD in planning [61,62], cognitive flexibility [63,64], and verbal fluency [65–67], while other studies did not find any differences between patients with ADHD and healthy controls in these cognitive functions [68–71]. Cognitive heterogeneity in adult ADHD is well recognized, meaning that most studies report cognitive impairments in adults with ADHD, but they vary in the type and degree of impairments. This heterogeneity may be attributed to variation within the ADHD group in the type and number of comorbid disorders, and medication status (e.g., in the present study, 36 patients with ADHD had comorbid disorders and 19 of them were taking medication for ADHD and/or mood disorders). It may be worth noting that errors and rule breaks were not considered when calculating test scores on the TOL and TMT tests. Errors and rule breaks provide information about inefficient strategy use, which is prevalent in several psychiatric disorders [53,54]. A study by Riccio et al. [69] showed that number of rule breaks on the TOL was associated with other executive functions and level of task difficulty in adults with ADHD, while other aspects of task performance such as number of correct responses were not.

Remarkably, our study found similar reaction time variability between the groups, which seems odd, as most of the previous studies suggest reaction time variability as a promising endophenotype for ADHD [17,72]. Kofler et al. [15] indicated that factors such as age play a role in reaction time variability. That is to say, reaction time variability is more pronounced in children compared to adults with ADHD. Reaction time variability may be sensitive to task characteristics such as inter-stimulus interval, more specifically longer inter-stimulus intervals elicit greater variability. The WAFV had very short inter-stimulus interval (500 ms.). This may have been too short for the differences to become evident. Another factor that might diminish reaction time variability is the use of stimulant treatments [15], however stimulant treatment did not likely influence the outcomes because only five participants took stimulant medication.

The present study also showed that the contribution of complex functions to variance in ADHD diagnosis when basic functions are not considered was 25%. This contribution dropped to 9% when basic functions are considered. This 64% reduction in variance in ADHD explained by complex processes when basic processes are already considered demonstrates the importance of basic processes for complex functions. This main finding replicates the study of Butzbach and colleagues [25] using a different selection of neuropsychological tests on an independent sample. The finding confirms previous reports, emphasizing the role of basic cognitive functions in ADHD symptomatology [26,27,30]. The study suggests that more caution should be taken when drawing conclusion about complex cognitive dysfunctions in ADHD without a proper investigation of basic functions. It also calls for future studies to test different task parameters that can differentiate between basic and complex functions in ADHD [73] not only for research but also for clinical assessment.

The present study emphasizes the role of basic processes on more complex cognitive functions in adult ADHD. The study is also in line with findings from other studies using different approaches such as applying a diffusion model in a reaction time task in children with ADHD. The diffusion model was used to differentiate between decisional (complex functions) and non-decisional time (basic functions) by analyzing the distribution of reaction time for both correct and incorrect responses as well as response accuracy [26,27]. This suggests that in adult ADHD the effects of basic processes on cognitive impairments can be considered robust, as it has been replicated using different methods to analyze task performance.

Our study may contribute to an ongoing discussion on the conceptualization of ADHD and whether the disorder is more related to bottom-up, top-down cognitive deficiencies, or their interplay. Several studies discussed that bottom-up processing speed [74] and distractibility [75] are highly influenced by a motivational deficit in ADHD. For example, findings of previous studies indicated that reaction time and omission errors are associated with reward and increasing boredom. It should be noted that tasks measuring basic cognitive function are usually long and repetitive which may decrease the motivation of subjects with ADHD. The present study did not control for the motivation effects and future studies may benefit from controlling for such effects.

### Limitations and future research

The significant group differences in the educational attainment could have influenced the outcomes. In the German educational system, educational levels are not truly hierarchical in nature. One might speculate that larger group differences in simple and complex cognitive functions become evident in a sample of adults with ADHD that have lower educational attainments compared to healthy controls. Having said that, our tested groups showed similar IQs, suggesting that educational attainment may have had only a limited influence. The educational level was not included in hierarchical regression model because adding the educational level as a nominal variable in the model leads to difficulties to interpret the outcomes.

Given the relevance of processing speed and distractibility, it may worthwhile for future studies to explore whether deficiencies in these functions occur during early perceptual processing or during the execution of motor response. It must be noted that a substantial number of patients with ADHD were diagnosed with one or more comorbid disorders, which may confound the conclusions drawn from this study. However, it can be argued that having comorbid disorders is typical for the nature of ADHD and can be attributed to the heterogeneity of this population [76–78].

### Conclusion

The present study replicated the findings of Butzbach and colleagues [25], confirming the significant contribution of basic processes to cognitive functions in adult ADHD. The study suggests that a proper investigation of basic functions would be valuable before drawing conclusion about complex cognitive dysfunctions in ADHD. Future studies are guided to test in depth whether deficiency in basic functions occurs at a perceptual or a motor execution phase of information processing.
